# DIRECT CARE WORKERS IN LTSS: RESEARCH AND POLICY AGENDA SETTING

**DOI:** 10.1093/geroni/igae098.1986

**Published:** 2024-12-31

**Authors:** Philip Taylor, Kezia Scales

**Affiliations:** University of Warwick, Coventry, England, United Kingdom; PHI, New York, New York, United States

## Abstract

At a time when the provision of long-term care is under more scrutiny than ever, and as demand for care increases, it is timely to consider what this will mean for the future organization of care work. Recent reviews have recommended changing the nature of the modern workplace, creating good work, and reducing poor-quality work. As well as positive worker outcomes, good work benefits organizational performance—and enhances care quality. The task is to develop a “conducive model” of jobs that generates positive outcomes for a care workforce that is disproportionately female and people of color who face multiple structural inequities. Drawing on international literature concerning the relationship between long-term care organization, staffing and care quality, the working environment, and worker and resident/client outcomes, this paper utilizes the multidimensional job quality concept to assess the state of knowledge. Unsurprisingly, the evidence demonstrates that poor-quality jobs result in poorer worker outcomes, and good-quality jobs support good care. However, significant gaps in understanding across dimensions of job quality are identified, particularly how it can be operationalized in day-to-day practice. It is argued that focusing on job quality will help reframe the analysis and interventions needed to improve care and adapt provision to meet future demand. In conversation with our worker and union leader guests and the panel scholars, we will collaboratively set the research and policy agenda for direct care workers in LTSS. Implications for establishing a national taskforce or an international concord on the direct care workforce will be discussed.

